# Independent and Supra-Additive Effects of Alcohol Consumption, Cigarette Smoking, and Metabolic Syndrome on the Elevation of Serum Liver Enzyme Levels

**DOI:** 10.1371/journal.pone.0063439

**Published:** 2013-05-07

**Authors:** Eun Young Park, Min Kyung Lim, Jin-Kyoung Oh, Heeyoun Cho, Mi Jin Bae, E. Hwa Yun, Dong-il Kim, Hai-Rim Shin

**Affiliations:** 1 National Cancer Control Institute, National Cancer Center, Goyang, Republic of Korea; 2 Department of Preventive Medicine, Seoul National University College of Medicine, Seoul, Republic of Korea; 3 Department of Occupational and Environmental Medicine, Samsung Medical Center, Sungkunkwan University School of Medicine, Suwon, Republic of Korea; 4 Non Communicable Diseases and Health Promotion, World Health Organization Western Pacific Regional Office, Manila, The Philippines; Bambino Gesu’ Children Hospital, Italy

## Abstract

We investigated the independent and combined effects of alcohol consumption, cigarette smoking and metabolic syndrome on abnormal liver function, i.e., the elevation of serum liver enzyme levels. Participants of a Korean population-based prospective cohort aged ≥30 years without liver disease, diabetes, or cardiovascular diseases were included. Information on alcohol consumption, smoking status, and metabolic syndrome, defined as per the criteria of the Adult Treatment Panel III, were applied to evaluate their impact on serum levels of aspartate aminotransferase (AST), alanine aminotransferase (ALT), and gamma-glutamyl transferase (GGT). Alcohol consumption, cigarette smoking and metabolic syndrome were the significant individual factors that elevated serum liver enzyme levels. Supra-additive effects of metabolic syndrome and either alcohol consumption or cigarette smoking were also identified. The combination of heavy drinking (≥24 g/day) and metabolic syndrome conferred an effect that was higher than the sum of the two individual effects (Synergic Index (SI): AST, 2.37 [1.20–4.67]; GGT, 1.91 [1.17–3.13]). Only GGT level (odds ratio 6.04 [3.68–9.94], SI 2.33 [1.24–4.41]) was significantly elevated when the effect of moderate drinking (<24 g/day) and metabolic syndrome was combined. The combined effect of any level of alcohol consumption and cigarette smoking was also supra-additive on the elevation of GGT level with SIs of 5.57 for drinking <24 g/day and smoking ≤20 pack years, 5.12 for <24 g/day and >20 pack years, 1.80 for ≥24 g/day and ≤20 pack years, 2.03 for ≥24 g/day and >20 pack years, while only the combined effect of drinking ≥24 g/day and smoking >20 pack years elevated the AST level (SI 4.55 [3.12–6.61]). The combined effect of cigarette smoking and metabolic syndrome was not supra-additive. To prevent fatty liver disease and other related diseases, a multifactorial prevention strategy that includes limited alcohol consumption, smoking cessation and rectification of adverse metabolic profiles is required.

## Introduction

The prevalence of unexplained elevated serum liver enzyme levels ranged from 2.8% to 5.4% of the general population in previous reports from the United States [1], [2]. Between 80% and 90% of the unexplained hypertransaminasemia are assumed to be representative of non-alcoholic fatty liver disease (NAFLD), which refers to a broad spectrum of disorders, from simple fatty liver disease to non-alcoholic steatohepatitis (NASH) with various degrees of fibrosis or intrahepatic necroinflammation. The prevalence of NAFLD was reported to be 25% in Italy [3], [4], 31% in the United States [5], 2–44% in the Europe [6], and 15–45% in Asia(China, Hong Kong, South Asia, South-East Asia, Korea, Japan and Taiwan) [7].

Previous studies have suggested that increased liver enzymes levels may also predict type 2 diabetes and cardiovascular disease independent of other potential risk factors, such as obesity and alcohol consumption [8], [9], [10]. In addition, a prospective Japanese study suggested that NAFLD might be a better predictor of cardiovascular disease than metabolic syndrome [11]. NAFLD is also considered to be a major cause of chronic liver disease and cryptogenic cirrhosis in developed countries [12], [13]. Features of metabolic syndrome, such as adiposity, NAFLD, hypertension, hyperglycemia and dyslipidemia may modulate liver fibrosis (i.e., NASH) through hepatic stellate cell activation stimulated by advanced glycation end product, macrophage infiltration, promotion of inflammation, leptin, angiotensin II, and oxidative stress [14]. Moreover, NAFLD and NASH have attracted attention for their associations with end-stage liver disease and hepatocellular carcinoma [15].

Metabolic syndrome is an important public health issue, because it known to be related to atherosclerosis, cardiovascular disease, increased cardiovascular disease mortality rates, and type 2 diabetes [16], [17], [18]. In previous studies, metabolic syndrome and obesity were strongly associated with abnormal liver function (i.e., elevated liver enzyme levels: aspartate aminotransferase [AST], alanine aminotransferase [ALT] and gamma-glutamyl transferase [GGT]) independent of hepatitis virus infection [19], [20].

Alcohol consumption is prevalent in many countries, and is one of the factors most frequently associated with elevated liver enzyme levels and alcohol-induced liver diseases, such as hepatic steatosis. Although there have been many studies on the relationship between alcohol consumption and liver damage, the subject is still under debate. In previous reports, 30 g of ethanol per day was proposed as a risk threshold for developing alcohol-induced hepatic damage, with a dose-response relationship, i.e., a risk that increases with increasing daily consumption [21]. In addition, moderate drinkers (defined as those who drank <40 g/day of alcohol) were found to have elevated liver enzyme levels compared to non-drinkers, and this difference was more apparent among men and the overweight or obese [22]. On the other hand, there are a few findings that suggested a protective effect of alcohol consumption on hepatic steatosis. Some studies reported that light (40–140 g/week) and moderate (140–280 g/week) alcohol consumption decreased the prevalence of fatty liver disease in the Japanese population [23], [24]. Furthermore, Hiramine and colleagues reported that consistent alcohol consumption (≥21 days/month) might contribute to a decrease in the prevalence of fatty liver disease [25]. Controvertible previous findings notwithstanding, the histological features of alcohol-induced hepatic steatosis are identical to those of NAFLD, although the pathogenic mechanisms that trigger hepatic fat accumulation are different. However, once steatosis occurs in the liver, similar mechanisms seem to be active in both alcoholic fatty liver disease (AFLD) and NAFLD [26]. Thus, it appears reasonable to conclude that alcohol consumption and metabolic profiles might have a combined effect on liver damage.

Cigarette smoking is considered a major cause of preventable morbidity and mortality worldwide. The major clinical consequences of cigarette smoking are chronic respiratory diseases, increased development of a variety of cancers, and increased risk of cardiovascular disease [27]. Although smoking is not considered a causative agent for chronic liver disease, there is increasing evidence that smoking may negatively impact the incidence, severity, and clinical course of many types of chronic liver diseases, including the development of hepatocellular carcinoma [28], [29], [30], [31].

In this context, a comprehensive investigation of the role of, and the relationship between alcohol consumption, cigarette smoking and metabolic profiles associated with elevated liver enzyme levels is necessary to understand the mechanisms of development of liver disease and to suggest an appropriate prevention plan.

## Materials and Methods

### Study Participants and Data Collection

The Korean National Cancer Center Cohort (KNCCC) is a population-based prospective cohort study conducted in rural and urban areas of South Korea. From July 2003 to July 2010 a total of 7,857 men and women residing in the counties of Haman and Sancheong, and the cities of Chuncheon and Changwon, which are areas of high liver cancer incidence and mortality, accepted to participate in the KNCCC. We restricted our analyses to the 5,946 men and women who reported at enrollment that they did not have liver disease, diabetes, or cardiovascular disease, were negative for hepatitis B surface antigen and hepatitis C virus antibody, and had a fasting blood sugar (FBS) lower than 126 mg/dL.

Well-trained interviewers conducted face-to-face interviews with participants using a structured questionnaire, which elicited information on various demographic characteristics: gender, age (30–39, 40–49, 50–59, 60–69, ≥70 years), education level (illiterate, elementary school or less, middle school, high school, college or more), average household income (10,000 KRW/month <50, 50–149, 150–299, ≥300), marital status (unmarried, married, divorced/widowed/separated), smoking status, alcohol consumption, history of exposure to pesticides (yes/no), medical history, family history of cancer, dietary factors, physical activity, occupational history, history of medication use, and reproductive history for women.

Alcohol consumption (g/day) was calculated from reported drinking frequency, volume, and ethanol content of alcoholic beverages consumed, and participants were subsequently categorized as non-drinkers (0 g/day), moderate drinkers (<24 g/day) and heavy drinkers (≥24 g/day). These cutoffs were defined as per the literature [32]. Participants were also classified according to their smoking status, as non-smokers, moderate smokers (pack year≤20), or heavy smokers (>20 pack years). Body mass index (BMI) was calculated from height and weight measurements taken at enrollment, and categorized according to the World Health Organization standards for Asians as follows: normal (<23 kg/m^2^), overweight (≥23 kg/m^2^ to <25 kg/m^2^) and obese (≥25 kg/m^2^) [33], [34].

Blood samples were collected after a fasting duration of 8 hours, and used to measure serum enzyme levels. AST and ALT levels were classified as normal for results of <40 U/L, and elevated for results of ≥40 U/L; and GGT as normal for results of <30 U/L and elevated for results of ≥30 U/L.

The study was approved by the Institutional Review Board of the National Cancer Center of Korea, and written informed consent was obtained from all study participants.

### Metabolic Syndrome Criteria

In this study, the diagnostic criteria set out in the Third Report of the National Cholesterol Education Program Expert Panel on Detection, Evaluation, and Treatment of High Blood Cholesterol in Adults (Adult Treatment Panel III) for metabolic syndrome were adopted using the abdominal obesity criterion modified for the Asian population [35]. According to the diagnostic criteria defined by the above-mentioned panel, a diagnosis of metabolic syndrome is rendered when more than three of the following five conditions occur simultaneously 1) waist circumference >102 cm for men, and >88 cm for women; 2) triglyceride level ≥150 mg/dL; 3) high-density lipoprotein (HDL) cholesterol level of <40 mg/dL for men, and <50 mg/dL for women; 4) blood pressure, ≥130 mmHg systolic or ≥85 mmHg diastolic; 5) impaired glucose tolerance manifested by FBS ≥110 mg/dL. In the present study BMI ≥25 kg/m^2^ was used as an indicator of obesity instead of waist circumference, since BMI ≥25 kg/m^2^ has been shown to predict cardiovascular disease risk in the Korean population [34].

### Statistical Analysis

We adjusted for age, gender, alcohol consumption, smoking status, education level, and average household income, which were considered potential confounding variables. We conducted multiple logistic regression analyses to investigate the relationship between alcohol consumption, cigarette smoking, metabolic syndrome, and liver enzyme levels. Statistical models were selected using a backward model selection strategy to reduce confounders from the initial model, which included all potential confounders. The adequacy of the model was determined using adjusted R^2^ in each model. Statistical analyses were performed using SAS (version 9.1). All statistical significance testing was two-sided with an α-error of 0.05.

To investigate the possible supra-additive effect of metabolic syndrome and alcohol consumption or cigarette smoking, the synergy index (SI) was calculated, using the methodology of Andersson et al [36]. A SI greater than one indicated a supra-additive effect.

## Results

Demographic and other selected characteristics of the study participants are presented in Table 1. Of the 5,946 study participants, 35.6% were men and 64.4% were women. A majority of study participants were aged 50 years or older and had an education level of elementary school or less. Statistically significant differences by gender were observed in the general characteristics of the study population and in the distribution of the components of metabolic syndrome. Women were more often obese (34.4%) and had a lower education level (illiterate or elementary school or less: 75.4%) than men, and most were non-smokers (90.4%) and non-drinkers (74.9%). Approximately 76% of participants had at least one component of metabolic syndrome (men: 77.2%, women: 74.7%) (Table 2).

**Table 1:** General characteristics of study participants by gender.

Characteristic		MenN (%)	WomenN (%)	TotalN (%)	P value
		2114 (35.6)	3832 (64.4)	5946 (100.0)	
Age group (years)	30–39	76 (3.6)	218 (5.7)	294 (4.9)	0.0036^a^
	40–49	274 (13.0)	535 (14.0)	809 (13.6)	
	50–59	504 (23.8)	837 (21.8)	1341 (22.6)	
	60–70	770 (36.4)	1450 (37.8)	2220 (37.3)	
	≥70	490 (23.2)	792 (20.7)	1282 (21.6)	
Education level	Illiterate	255 (12.1)	1259 (32.9)	1514 (25.5)	<0.0001^a^
	Elementary school or less	870 (41.2)	1628 (42.5)	2498 (42.0)	
	Middle school	410 (19.4)	410 (10.7)	820 (13.8)	
	High school	388 (18.4)	374 (9.8)	762 (12.8)	
	College or more	172 (8.1)	120 (3.1)	292 (4.9)	
	Missing	19 (0.9)	41 (1.1)	60 (1.0)	
Average household income (10,000 KRW/month)	<50	789 (37.3)	1896 (49.5)	2685 (45.2)	0.0497^a^
	50–149	630 (29.8)	930 (24.3)	1560 (26.2)	
	150–299	399 (18.9)	508 (13.3)	907 (15.3)	
	≥300	164 (7.8)	192 (5.0)	356 (6.0)	
	Missing	132 (6.2)	306 (8.0)	438 (7.4)	
Marital status	Unmarried	28 (1.3)	15 (0.4)	43 (0.7)	<0.0001^a^
	Married	1938 (91.7)	2526 (65.9)	4464 (75.1)	
	Divorced/widowed/separated	124 (5.9)	1248 (32.6)	1372 (23.1)	
	Missing	24 (1.1)	43 (1.1)	67 (1.1)	
Smoking status (pack years)	Non-smokers	406 (19.4)	3463 (91.9)	3869 (66.0)	<0.0001^a^
	≤20 pack years	525 (25.1)	222 (5.9)	747 (12.8)	
	>20 pack years	1061 (50.7)	55 (1.5)	1116 (19.0)	
	Missing	100 (4.8)	28 (0.7)	128 (2.2)	
Alcohol consumption	Non-drinkers	694 (32.8)	2869 (74.9)	3563 (59.9)	<0.0001^a^
	Moderate (<24 g/day)	536 (25.4)	571 (14.9)	1107 (18.6)	
	Heavy (≥24 g/day)	641 (30.3)	71 (1.9)	712 (12.0)	
	Missing	243 (11.5)	321 (8.4)	564 (9.5)	
History of exposure to pesticides	No	396 (18.7)	1364 (35.6)	1760 (29.6)	<0.0001^a^
	Yes	1695 (80.2)	2408 (62.8)	4103 (69.0)	
	Missing	23 (1.1)	60 (1.6)	83 (1.4)	

a Mantel-Haenszel chisquare test.

**Table 2:** Distribution of each component of metabolic syndrome and serum liver enzyme level by gender.

Characteristic		MenN (%)	WomenN (%)	TotalN (%)	P value
		2114 (35.6)	3832 (64.4)	5946 (100.0)	
BMI (kg/m^2^)	<23	1066 (50.4)	1593 (41.6)	2659 (44.7)	<0.0001^a^
	≥23 to <25	527 (24.9)	920 (24.0)	1447 (24.3)	
	≥25	521 (24.7)	1319 (34.4)	1840 (31.0)	
FBS (mg/dL)	<110	1993 (94.3)	3654 (95.4)	5647 (95.0)	0.0685^b^
	≥110	121 (5.7)	178 (4.7)	299 (5.0)	
Triglyceride level (mg/dL)	<150	1459 (69.0)	2768 (72.2)	4227 (71.1)	0.0088^b^
	≥150	655 (31.0)	1064 (27.8)	1719 (28.9)	
HDL cholesterol level	≥40 (≥50 for women)	1753 (82.9)	3353 (87.5)	5106 (85.9)	<0.0001^b^
	<40 (<50 for women)	361 (17.1)	479 (12.5)	840 (14.1)	
Blood pressure^c^	Normal	969 (45.8)	1825 (47.6)	2794 (47.0)	0.1861^b^
	Abnormal	1145 (54.2)	2007 (52.4)	3152 (53.0)	
No. of components of metabolic syndrome	0	481 (22.8)	969 (25.3)	1450 (24.4)	<0.0001^a^
	1	779 (36.9)	1480 (38.6)	2259 (38.0)	
	2	579 (27.4)	1027 (26.8)	1606 (27.0)	
	3	230 (10.9)	311 (8.1)	541 (9.1)	
	4	43 (2.0)	44 (1.2)	87 (1.5)	
	5	2 (0.1)	1 (0.0)	3 (0.1)	
Metabolic syndrome	No	1839 (87.0)	3476 (90.7)	5315 (89.4)	<0.0001^b^
	Yes	275 (13.0)	356 (9.3)	631 (10.6)	
AST (U/L)	<40	1915 (90.6)	3735 (97.5)	5650 (95.0)	<0.0001^b^
	≥40	199 (9.4)	97 (2.5)	296 (5.0)	
ALT (U/L)	<40	1992 (94.2)	3763 (98.2)	5755 (96.8)	<0.0001^b^
	≥40	122 (5.8)	69 (1.8)	191 (3.2)	
GGT (U/L)	<30	1399 (66.2)	3620 (94.5)	5019 (84.4)	<0.0001^b^
	≥30	715 (33.8)	212 (5.5)	927 (15.6)	

a Mantel-Haenszel chisquare test.

b Chisquare test.

c Normal: systolic <130 mmHg and diastolic <85 mmHg; abnormal: systolic ≥130 mmHg or diastolic ≥85 mmHg.

AST: aspartate aminotransferase, ALT: alanine aminotransferase, BMI: body mass index, FBS: fasting blood glucose, GGT: gamma-glutamyl transferase, HDL: high-density lipoprotein, KRW: Korean Won.

Heavy smokers (>20 pack years) were older and had a lower education level (illiterate or elementary school or less: 59.3%) and lower average income (<150 KRW/month: 76.8%). Moderate drinkers (<24 g/day) had a higher education level (high school more: 31.8%) and higher average income (>150 KRW/month: 35.2%). When we explored factors associated with metabolic syndrome, moderate drinking (<24 g/day) had a protective effect, while heavy alcohol consumption (≥24 g/day) did not have any significant association. A college education level or higher was positively associated with metabolic syndrome among men only. No significant association was observed between cigarette smoking and metabolic syndrome (data not shown).

Table 3 presents the associations of elevated liver enzyme levels with metabolic syndrome, alcohol consumption and cigarette smoking. The adjusted odds of having elevated AST, ALT and GGT were significantly higher in participants with, than participants without metabolic syndrome. For alcohol consumption, the adjusted odds of having elevated AST, ALT and GGT were higher in heavy drinkers (≥24 g/day). In moderate drinkers (<24 g/day) only GGT was elevated. In addition, heavy smokers (pack years>20) had higher risk of elevated AST and GGT. After stratification by gender, the relationship was stronger in men, while it either weakened or disappeared in women (Table 3).

**Table 3:** Association of elevated liver enzyme levels with metabolic syndrome, alcohol consumption and smoking status, Korea National Cancer Center Cohort.

			AST		ALT		GGT	
			Prevalence (%)	Odds ratio (95% CI)	Prevalence (%)	Odds ratio (95% CI)	Prevalence (%)	Odds ratio (95% CI)
Overall	Metabolic syndrome	No (risk<3)	243 (4.6)	1.00	150 (2.8)	1.00	759 (14.3)	1.00
		Yes (risk≥3)	53 (8.4)	1.69 (1.23–2.32)	41 (6.5)	2.23 (1.55–3.21)	168 (26.6)	2.05 (1.65–2.54)
	Alcohol consumption	Non-drinkers	93(2.6)	1.00^b^	78 (2.2)	1.00^a^	204 (5.7)	1.00^b^
		Moderate (<24 g/day)	50 (4.5)	1.43 (0.99–2.07)	32 (2.9)	0.88 (0.56–1.37)	221 (20.0)	2.76 (2.22–3.44)
		Heavy (≥24 g/day)	113 (15.9)	4.21 (2.95–6.00)	56 (7.9)	1.86 (1.22–2.85)	381 (53.5)	8.05 (6.38–10.16)
	Smoking status (pack years)	Non-smokers	107 (2.8)	1.00^a^	83 (2.2)	1.00^a^	282 (7.3)	1.00^b^
		≤20 pack years	50 (6.7)	1.38 (0.87–2.17)	32 (4.3)	1.04 (0.60–1.79)	195 (26.1)	1.42 (1.07–1.88)
		>20 pack years	120 (10.8)	1.89 (1.22–2.92)	64 (5.7)	1.28 (0.76–2.15)	400 (35.84)	1.70 (1.29–2.23)
Men	Metabolic syndrome	Negative (risk<3)	154 (8.4)	1.00	88 (4.8)	1.00	579 (31.5)	1.00
		Positive (risk≥3)	45 (16.4)	2.04 (1.41–2.93)	34 (12.4)	2.67 (1.74–4.08)	136 (49.5)	2.04 (1.57–2.66)
	Alcohol consumption	Non-drinkers	25 (3.6)	1.00^b^	26 (3.8)	1.00^a^	74 (10.7)	1.00^b^
		Moderate (<24 g/day)	36 (6.7)	1.93 (1.13–3.27)	24 (4.5)	1.03 (0.58–1.83)	182 (34.0)	4.14 (3.05–5.62)
		Heavy (≥24 g/day)	109 (17.0)	5.16 (3.28–8.12)	55 (8.6)	2.05 (1.26–3.34)	366 (57.1)	10.67 (7.98–14.28)
	Smoking status (pack years)	Non-smokers	21(5.2)	1.00^a^	16 (3.9)	1.00^a^	103 (25.4)	1.00^a^
		≤20 pack years	42 (8.0)	1.43 (0.79–2.58)	31 (5.9)	1.35 (0.69–2.64)	176 (33.5)	1.29 (0.92–1.81)
		>20 pack years	120 (11.3)	2.08 (1.23–3.53)	64 (6.0)	1.57 (0.86–2.89)	395 (37.2)	1.64 (1.22–2.22)
Women	Metabolic syndrome	Negative (risk<3)	89 (2.6)	1.00	62 (1.8)	1.00	180 (5.2)	1.00
		Positive (risk≥3)	8 (2.3)	0.85 (0.41–1.78)	7 (2.0)	1.20 (0.54–2.67)	32 (9.0)	1.85 (1.24–2.75)
	Alcohol consumption	Non-drinkers	68 (2.4)	1.00	52 (1.8)	1.00	130 (4.5)	1.00^b^
		Moderate (<24 g/day)	14 (2.5)	1.05 (0.58–1.90)	8 (1.4)	0.71 (0.33–1.52)	39 (6.8)	1.61 (1.10–2.34)
		Heavy (≥24 g/day)	4 (5.6)	2.51 (0.89–7.09)	1 (1.4)	0.84 (0.11–6.20)	15 (21.1)	5.68 (3.12–10.34)
	Smoking status (pack years)	Non-smokers	86 (2.5)	1.00	67 (1.9)	1.00	179 (5.2)	1.00
		≤20 pack years	8 (3.6)	1.53 (0.72–3.25)	1 (0.5)	0.30 (0.04–2.19)	19 (8.6)	1.62 (0.95–2.77)
		>20 pack years	0 (0.0)	–	0 (0.0)	–	5 (9.1)	1.76 (0.67–4.61)

a p for trend<0.05,

b p for trend<0.0001.

NOTE: Metabolic syndrome model was adjusted for age, gender, alcohol consumption, smoking status and education level. Alcohol consumption model was adjusted for age, gender, smoking status (pack years), metabolic syndrome and education level. Smoking status model was adjusted for age, gender, alcohol consumption, metabolic syndrome and education level.

AST: aspartate aminotransferase, ALT: alanine aminotransferase, CI: confidence interval, GGT: gamma-glutamyl transferase.

Table 4 shows the adjusted odds of having elevated liver enzyme levels by metabolic profile. Odds were significantly higher in participants with high FBS (≥110 mg/dL), high triglyceride level (≥150 mg/dL) or abnormal blood pressure (≥130 mmHg systolic or ≥85 mmHg diastolic). On the other hand, HDL cholesterol level had an inverse association with liver enzyme levels, and BMI had no association. The number of components of metabolic syndrome that occurred simultaneously in a participant showed a strong dose-response relationship with AST (p<0.0001), ALT (p<0.0001) and GGT (p<0.0001) levels. Indeed, liver enzyme levels increased even among participants who had only one component of metabolic syndrome. After stratification by gender, similar relationships were found in men, but were either weaker or non-existent in women (Table 5, 6).

**Table 4:** Associations of metabolic profile and number of components of metabolic syndrome with elevated liver enzyme levels, Korean National Cancer Center Cohort (Overall).

		AST		ALT		GGT	
		Prevalence (%)	Odds ratio (95% CI)	Prevalence (%)	Odds ratio (95% CI)	Prevalence (%)	Odds ratio (95% CI)
FBS (mg/dL)							
	<110	272 (4.8)	1.00	174 (3.1)	1.00	842 (14.9)	1.00
	≥110	24 (8.0)	1.51 (0.96–2.38)	17 (5.7)	1.82 (1.07–3.09)	85 (28.4)	2.29 (1.68–3.13)
Triglyceride level (mg/dL)							
	<150	160 (3.8)	1.00	96 (2.3)	1.00	494 (11.7)	1.00
	≥150	136 (7.9)	1.92 (1.5–2.46)	95 (5.5)	2.12 (1.57–2.87)	433 (25.2)	2.67 (2.26–3.16)
HDL cholesterol level (mg/dL)							
	≥40 (≥50 for women)	252 (4.9)	1.00	160 (3.1)	1.00	810 (15.9)	1.00
	<40 (<50 for women)	44 (5.2)	0.83 (0.59–1.18)	31 (3.7)	0.96 (0.64–1.44)	117 (13.9)	0.55 (0.44–0.70)
Blood pressure^a^							
	Normal	93 (3.3)	1.00	58 (2.1)	1.00	332 (11.9)	1.00
	Abnormal	203 (6.4)	1.83 (1.41–2.37)	133 (4.2)	2.05 (1.48–2.84)	595 (18.9)	1.66 (1.41–1.96)
BMI (kg/m2)							
	<25	236 (5.3)	1.00	146 (3.3)	1.00	691 (15.4)	1.00
	≥25	60 (4.2)	0.73 (0.54–0.98)	45 (3.1)	0.86 (0.60–1.21)	236 (16.3)	0.98 (0.81–1.17)
Number of metabolic syndrome components							
	0	46 (3.2)	1.00	21 (1.5)	1.00	139 (9.6)	1.00
	1	97 (4.3)	1.35 (0.94–1.94)	67 (3.0)	2.21 (1.34–3.65)	305 (13.5)	1.52 (1.21–1.90)
	2	100 (6.2)	1.94 (1.35–2.78)	62 (3.9)	2.81 (1.70–4.66)	315 (19.6)	2.41 (1.92–3.04)
	3	42 (7.8)	2.26 (1.46–3.50)	34 (6.3)	4.50 (2.57–7.91)	141 (26.1)	3.28 (2.46–4.37)
	4	11 (12.6)	3.57 (1.74–7.33)	7 (8.1)	5.32 (2.15–13.17)	27 (31.0)	3.66 (2.10–6.38)
	5	0 (0.0)	**–**	0 (0.0)	**–**	0 (0.0)	**–**
			<0.0001^b^		<0.0001^b^		<0.0001^b^

a Normal: systolic <130 mmHg and diastolic <85 mmHg. Abnormal: systolic ≥130 mmHg or diastolic ≥85 mmHg.

b p for trend.

NOTE: No. of components of metabolic syndrome models were adjusted for age, gender, alcohol consumption, smoking status and education level. Components of metabolic syndrome were adjusted age, gender, FBS, triglyceride level, HDL cholesterol level, blood pressure, BMI, alcohol consumption, smoking status and education level.

AST: aspartate aminotransferase, ALT: alanine aminotransferase, BMI: body mass index, CI: confidence interval, FBS: fasting blood glucose, GGT gamma-glutamyl transferase, HDL: high-density lipoprotein.

**Table 5:** Associations of metabolic profile and number of components of metabolic syndrome with elevated liver enzyme levels, Korean National Cancer Center Cohort (Men).

		AST		ALT		GGT	
		Prevalence (%)	Odds ratio (95% CI)	Prevalence (%)	Odds ratio (95% CI)	Prevalence (%)	Odds ratio (95% CI)
FBS (mg/dL)							
	<110	181 (9.1)	1.00	111 (5.6)	1.00	654 (32.8)	1.00
	≥110	18 (14.9)	1.63 (0.93–2.83)	11 (9.1)	1.68 (0.85–3.34)	61 (50.4)	2.04 (1.36–3.07)
Triglyceride level (mg/dL)							
	<150	95 (6.5)	1.00	47 (3.2)	1.00	365 (25.0)	1.00
	≥150	104 (15.9)	2.41 (1.76–3.30)	75 (11.5)	2.99 (2.02–4.44)	350 (53.4)	3.26 (2.64–4.01)
HDL cholesterol level (mg/dL							
	≥40	168 (9.6)	1.00	101 (5.8)	1.00	617 (35.2)	1.00
	<40	31 (8.6)	0.74 (0.49–1.12)	21 (5.8)	0.82 (0.49–1.36)	98 (27.2)	0.52 (0.40–0.69)
Blood pressure^a^							
	Normal	59 (6.1)	1.00	31 (3.2)	1.00	247 (25.5)	1.00
	Abnormal	140 (12.2)	1.90 (1.37–2.64)	91 (8.0)	2.39 (1.55–3.69)	468 (40.9)	1.84 (1.51–2.25)
BMI (kg/m2)							
	<25	154 (9.7)	1.00	89 (5.6)	1.00	530 (33.4)	1.00
	≥25	45 (8.5)	0.79 (0.55–1.13)	33 (6.3)	0.95 (0.62–1.46)	185 (35.1)	0.93 (0.74–1.17)
Number of metabolic syndrome components							
	0	28 (5.8)	1.00	9 (1.9)	1.00	100 (20.8)	1.00
	1	59 (7.6)	1.31 (0.82–2.10)	35 (4.5)	2.55 (1.21–5.38)	226 (29.0)	1.56 (1.19–2.06)
	2	67 (11.6)	2.06 (1.30–3.28)	44 (7.6)	4.18 (2.01–8.70)	253 (43.7)	2.88 (2.18–3.82)
	3	35 (15.2)	2.75 (1.62–4.68)	28 (12.2)	7.01 (3.23–15.23)	114 (49.6)	3.61 (2.55–5.11)
	4	10 (23.3)	4.44 (1.96–10.05)	6 (14.0)	7.53 (2.50–22.63)	22 (51.2)	3.56 (1.85–6.85)
	5	0 (0.0)	**–**	0 (0.0)	**–**	0 (0.0)	**–**
			<0.0001^b^		<0.0001^b^		<0.0001^b^

a Normal: systolic <130 mmHg and diastolic <85 mmHg. Abnormal: systolic ≥130 mmHg or diastolic ≥85 mmHg.

b p for trend.

NOTE: No. of components of metabolic syndrome models were adjusted for age, gender, alcohol consumption, smoking status and education level. Components of metabolic syndrome were adjusted age, gender, FBS, triglyceride level, HDL cholesterol level, blood pressure, BMI, alcohol consumption, smoking status and education level.

AST: aspartate aminotransferase, ALT: alanine aminotransferase, BMI: body mass index, CI: confidence interval, FBS: fasting blood glucose, GGT gamma-glutamyl transferase, HDL: high-density lipoprotein.

**Table 6:** Associations of metabolic profile and number of components of metabolic syndrome with elevated liver enzyme levels, Korean National Cancer Center Cohort (Women).

		AST		ALT		GGT	
		Prevalence (%)	Odds ratio (95% CI)	Prevalence (%)	Odds ratio (95% CI)	Prevalence (%)	Odds ratio (95% CI)
FBS (mg/dL)							
	<110	91 (2.5)	1.00	63 (1.7)	1.00	188 (5.2)	1.00
	≥110	6 (3.4)	1.21 (0.52–2.84)	6 (3.4)	1.93 (0.81–4.59)	24 (13.5)	2.72 (1.71–4.33)
Triglyceride level (mg/dL)							
	<150	65 (2.4)	1.00	49 (1.8)	1.00	129 (4.7)	1.00
	≥150	32 (3.0)	1.19 (0.76–1.84)	20 (1.9)	1.07 (0.62–1.85)	83 (7.8)	1.72 (1.28–2.31)
HDL cholesterol level (mg/dL)							
	≥50	84 (2.5)	1.00	59 (1.8)	1.00	193 (5.8)	1.00
	<50	13 (2.7)	1.03 (0.57–1.89)	10 (2.1)	1.25 (0.63–2.50)	19 (4.0)	0.60 (0.37–0.97)
Blood pressure^a^							
	Normal	34 (1.9)	1.00	27 (1.5)	1.00	85 (4.7)	1.00
	Abnormal	63 (3.1)	1.65 (1.07–2.54)	42 (2.1)	1.53 (0.92–2.53)	127 (6.3)	1.29 (0.97–1.73)
BMI (kg/m2)							
	<25	82 (2.8)	1.00	57 (2.0)	1.00	161 (5.5)	1.00
	≥25	15 (1.6)	0.58 (0.33–1.00)	12 (1.3)	0.66 (0.35–1.23)	51 (5.5)	1.03 (0.74–1.43)
Number of metabolic syndrome components							
	0	18 (1.9)	1.00	12 (1.2)	1.00	39 (4.0)	1.00
	1	38 (2.6)	1.36 (0.77–2.41)	32 (2.2)	1.87 (0.95–3.68)	79 (5.3)	1.35 (0.91–2.00)
	2	33 (3.2)	1.68 (0.93–3.03)	18 (1.8)	1.59 (0.75–3.36)	62 (6.0)	1.52 (1.00–2.30)
	3	7 (2.3)	1.17 (0.48–2.86)	6 (1.9)	1.85 (0.68–5.06)	27 (8.7)	2.33 (1.39–3.91)
	4	1 (2.3)	1.17 (0.15–9.02)	1 (2.3)	2.12 (0.27–16.96)	5 (11.4)	3.18 (1.18–8.62)
	5	0 (0.0)	**–**	0 (0.0)	**–**	0 (0.0)	**–**
			0.2916^b^		0.2665^b^		0.0006^b^

a Normal: systolic <130 mmHg and diastolic <85 mmHg. Abnormal: systolic ≥130 mmHg or diastolic ≥85 mmHg.

b p for trend.

NOTE: No. of components of metabolic syndrome models were adjusted for age, gender, alcohol consumption, smoking status and education level. Components of metabolic syndrome were adjusted age, gender, FBS, triglyceride level, HDL cholesterol level, blood pressure, BMI, alcohol consumption, smoking status and education level.

AST: aspartate aminotransferase, ALT: alanine aminotransferase, BMI: body mass index, CI: confidence interval, FBS: fasting blood glucose, GGT gamma-glutamyl transferase, HDL: high-density lipoprotein.

**Figure 1:**
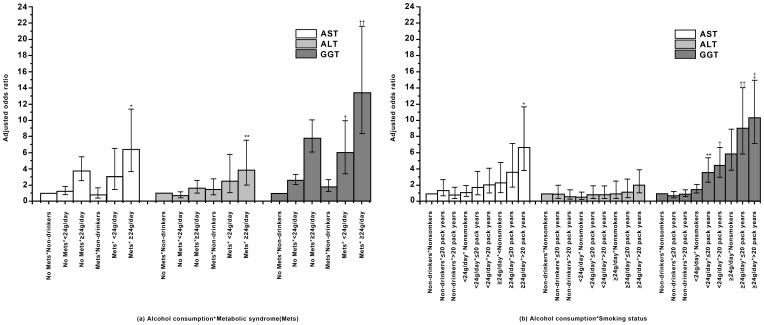
Supra-additive effect of alcohol consumption, cigarette smoking and metabolic syndrome on the elevation of liver enzyme levels. NOTES: Model of alcohol consumption x metabolic syndrome was adjusted for age, gender, education level and smoking status. Model of alcohol consumption × cigarette smoking was adjusted for age, gender, level of education, alcohol consumption, and metabolic syndrome. (a) The combined risk of heavy drinking (≥24 g/day) and metabolic syndrome was *AST: OR 6.42 (3.64–11.35), SI 2.37 (1.20–4.67); **ALT: OR 3.88 (2.00–7.55), SI 2.65 (0.80–8.76); ††GGT: OR 13.43 (8.35–21.60), SI 1.91 (1.17–3.13). The combined risk of moderate drinking (<24 g/day) and metabolic syndrome was shown only for ^†^GGT: OR 6.04 (3.68–9.94), SI 2.33 (1.24–4.41). (b) The combined risk of heavy drinking (≥24 g/day) and >20 pack years was *AST: OR 6.71 (3.85–11.69), SI 4.55 (3.12–6.61). For GGT, **moderate alcohol consumption (<24 g/day) and ≤20 pack years, OR 3.60 (2.38–5.43), SI 5.57 (1.63–19.06); ^†^<24 g/day and >20 pack years, OR 4.50 (3.04–6.67), SI 5.12 (2.19–12.00); ^††^≥24 g/day and ≤20 pack years, OR 9.04 (5.85–13.99), SI 1.80 (1.57–2.06); ^‡^≥24 g/day and >20 pack years, OR 10.31 (7.13–14.89), SI 2.03 (1.75–2.35). AST, aspartate aminotransferase; ALT, alanine aminotransferase; GGT, gamma-glutamyl transferase; OR, odds ratio; SI, synergy index.

Figure 1 depicts the supra-additive effect of alcohol consumption, cigarette smoking and metabolic syndrome on the elevation of liver enzyme levels. Heavy drinkers (≥24 g/day) without metabolic syndrome had a high risk of elevated liver enzyme levels (OR: AST: 3.73 [2.54–5.47], ALT: 1.61 [1.00–2.60], GGT: 7.81 [6.07–10.06]), as did moderate drinkers (<24 g/day) without metabolic syndrome (OR: GGT: 2.61[2.06–3.32]), demonstrating the independent risk conferred by alcohol consumption. An independent risk for metabolic syndrome among non-drinkers was found only for GGT (OR: 1.79 [1.21–2.66]) level. The risk of having elevated liver enzyme levels conferred by the combination of heavy drinking and metabolic syndrome was 6.42 [3.64–11.35] for AST, 3.88 [2.00–7.55] for ALT and 13.43 [8.35–21.60] for GGT, which were more than the sum of the two individual effects (SI: AST, 2.37 [1.20–4.67]; GGT, 1.91 [1.17–3.13]). In addition, the supra-additive effect of moderate alcohol consumption (<24 g/day) and metabolic syndrome was manifested in an elevated GGT level only (OR: 6.04 [3.68–9.94], SI: 2.33 [1.24–4.41]) (Figure 1). When analyzed separately by gender, the independent and synergistic effects of alcohol consumption and metabolic syndrome remained in men, but not in women (data not shown). No independent effect was observed for cigarette smoking among non-drinkers. However, the combined effect of alcohol consumption and cigarette smoking was supra-additive. The risk of having elevated liver enzyme levels conferred by the combination of heavy drinking (≥24 g/day) and heavy smoking (>20 pack years) was 6.71 [3.85–11.69] for AST (SI: 4.55 [3.12–6.61]). For GGT, the supra-additive effect of alcohol consumption and cigarette smoking was more prominent (OR: <24 g/day and ≤20 pack years, 3.60 [2.38–5.43]; <24 g/day and >20 pack years, 4.50 [3.04–6.67]; ≥24 g/day and ≤20 pack year, 9.04 [5.85–13.99]; ≥24 g/day and >20 pack years, 10.31 [7.13–14.89]), which was larger than the sum of the two individual effects (SI: <24 g/day and ≤20 pack years, 5.57 [1.63–19.06]; <24 g/day and >20 pack years, 5.12 [2.19–12.00]; ≥24 g/day and ≤20 pack years, 1.80 [1.57–2.06]; ≥24 g/day and >20 pack years, 2.03 [1.75–2.35]). On the other hand, the combined effect of metabolic syndrome and cigarette smoking was not supra-additive (data not shown).

We explored the combined effects of alcohol consumption, cigarette smoking and the individual components of metabolic syndrome on the elevation of liver enzyme levels (data not shown). The combined effect of triglyceride level ≥150 mg/dL and moderate alcohol consumption (<24 g/day) on GGT was supra-additive (SI: 2.90 [1.85–4.55]). Similar relationships were also revealed for abnormal blood pressure (SI: 2.37 [1.38–4.08]). Among heavy drinkers (≥24 g/day), a significant supra-additive effect was found (SI: triglyceride level, AST 3.02 [1.62–5.63]; GGT 2.70 [1.89–3.87]; blood pressure, AST 2.48 [1.33–4.67]; GGT 1.44 [1.01–2.05]). The combined effect of cigarette smoking and the individual components of metabolic syndrome was not supra-additive.

## Discussion

In this study, a supra-additive effect of alcohol consumption (even moderate alcohol consumption, <24 g/day) and both cigarette smoking and metabolic syndrome was observed on serum liver enzyme levels. Given the rapid increase in the prevalence of adverse metabolic profiles and the high prevalence of alcohol consumption and cigarette smoking, the present study suggests the need to develop strategic plans to prevent fatty liver disease and other related chronic diseases using a multifactorial approach that would integrate proactive behavioral changes, such as stricter control of alcohol consumption and cigarette smoking, including moderate drinking and smoking cessation, avoidance of obesity and increased physical activity to rectify adverse metabolic profiles.

It is known that alcohol consumption and components of metabolic syndrome elevate serum liver enzymes levels, as well as the risk of developing AFLD or NAFLD [2], [19], [20], [37]. Conversely, the association between cigarette smoking and serum liver enzyme levels and NAFLD has been sparsely reported and the mechanism of these associations has not been fully elucidated. Hamabe and colleagues reported that cigarette smoking is associated with the onset of NAFLD and elevates the risk of its development 1.91-fold [38]. In contrast, Breitling and colleagues reported that cigarette smoking by itself was unrelated to GGT level [39].

Some previous studies showed the combined risk of alcohol consumption and metabolic syndrome on liver enzyme levels and fatty liver disease [26], [40]. However, their results were not consistent and their magnitudes of risk and the supra-additive effect of these factors were not clear. Haren and colleagues reported that adverse metabolic risk cluster profile increased the risk in risky drinkers (>40 g/day in men, >20 g/day in women) than in nondrinkers by three-fold [40].Adams and colleagues found no synergistic effect between alcohol consumption and BMI or waist circumference on ALT or GGT levels [26]. Moreover, the risk of moderate alcohol consumption (<24 g/day) and its synergistic relationship with metabolic syndrome were not explored in most of the previous studies.

In this context, the present study provides evidence of a supra-additive effect of heavy alcohol consumption (≥24 g/day) and metabolic syndrome, as well as triglyceride level and abnormal blood pressure (a component of metabolic syndrome), on the elevation of liver enzyme levels (i.e., AST, GGT). Our study also suggests that moderate alcohol consumption (<24 g/day) alone may increase liver enzyme levels, and that the joint effects of moderate alcohol consumption and metabolic syndrome may be supra-additive on the elevation of GGT levels. Our findings showed that moderate alcohol consumption (<24 g/day) in combination with metabolic syndrome increased liver enzyme levels three- to six-fold. Moreover, metabolic syndrome with heavy alcohol consumption (≥24 g/day) increased liver enzyme levels four to 16-fold.

We also observed an effect of cigarette smoking, and moreover, a supra-additive effect of alcohol consumption and cigarette smoking on the elevation of serum liver enzyme levels in the present study. Few previous studies have addressed the interaction between alcohol consumption and cigarette smoking on the elevation of serum liver enzyme levels. Breitling and colleagues reported that moderate to heavy alcohol consumption (>100 g/week) was related to a 1.7-fold increased risk of elevated GGT levels (>50 IU/L) compared to individuals that did not drink or smoke, whereas cigarette smoking by itself was not associated with GGT levels. However, when moderate to heavy alcohol consumption was present in combination with heavy smoking, the risk increased 2.9-fold in women and 3.8-fold in men [39]. In addition, only Breitling and colleagues proposed the hypothesis that both alcohol consumption and cigarette smoking might induce depletion of oxidative stress defense substances, such as glutathione, in various tissues, and both substances had additive effects in their relationship [39].

We also confirmed previous results that metabolic syndrome and alcohol consumption were associated with elevated liver enzyme levels [3], [20], [21], [37], [41], [42]. The associations between alcohol consumption and elevated GGT levels were shown for both moderate alcohol drinking (<24 g/day), and heavy alcohol drinking (≥24 g/day), with a dose-response pattern, while the association between alcohol consumption and elevated ALT and AST levels was shown for heavy alcohol drinking (≥24 g/day) only. This corresponds to results from previous studies that suggested a protective effect of continuous moderate alcohol drinking on fatty liver disease. However, our present study did not link alcohol consumption to fatty liver disease, but to elevated liver enzyme levels. Moreover our findings suggested that even an alcohol consumption lower than that referred to in previous studies (i.e., lower than ≥30 g/day or ≥40 g/day) 21,40 may confer some risk.

This study showed that elevated liver enzyme levels were associated with a higher triglyceride level and abnormal blood pressure (i.e., components of metabolic syndrome), and had strong dose-response relationships with the number of metabolic syndrome components; meaning that even if the participants did not have metabolic syndrome, those with an adverse metabolic profile were still at higher risk of abnormal liver function than participants with no adverse metabolic profile. These results are consistent with previous studies. Bedogni et al. suggested that NAFLD was associated with BMI ≥30 kg/m^2^, triglyceride level ≥150 mg/dL; systolic blood pressure ≥130 mmHg and FBS ≥110 mg/dL [3]. Oh et al. also found that elevated ALT was significantly associated with increased triglyceride levels, FBS, BMI and diastolic blood pressure, and that the prevalence and ORs of elevated ALT level increased significantly with increasing number of components of metabolic syndrome [2]. However, HDL cholesterol level had an inverse association, and BMI had no association with liver enzyme levels in the present study. These results could be due to a relatively low prevalence of low HDL cholesterol levels in this study population, or misclassification of obesity due to use of BMI instead of waist circumference. In Asian populations, people with normal or lower BMI are likely to have central obesity, also referred to as metabolic obesity, and to suffer from NAFLD/NASH. Moreover, this study population showed discordance among adverse metabolic profiles (e.g., high triglyceride level and low HDL cholesterol level, abnormal blood pressure and obesity; discordance %: 29.72% for triglyceride level and HDL cholesterol level, and 37.67% for blood pressure and obesity), which might affect the association of HDL cholesterol level or BMI with liver enzyme levels.

The histological features of AFLD are known to be identical to those of NAFLD, although the pathogenic mechanisms that induce hepatic fat accumulation are different. However, once steatosis occurs in the liver, both AFLD and NAFLD pathogenesis seem to be substantially similar [43]. There are several mechanisms known to play central roles in the progression of liver disease, including increased oxidative stress, mitochondrial dysfunction, inflammation, hepatocellular apoptosis and fibrogenesis. Oxidative stress and mitochondrial dysfunction are almost always present in the pathogenesis of steatohepatitis, regardless of its initial cause. In addition, oxidative stress and mitochondrial dysfunction, when combined with insulin resistance, can have a synergistic effect, which causes chronic accumulation of free fatty acid in the liver, antioxidant depletion, increased cytokine-mediated hepatoxicity, and promotion of stellate cell activation and proliferation. This process ultimately leads to increased inflammation, apoptosis and liver fibrosis [43], [44], [45]. The present study adds to the existing literature, with meaningful and significant findings on the role of alcohol consumption, cigarette smoking and metabolic syndrome in the development of liver disease.

Several limitations should be taken into account when interpreting our results. First, this study had a cross-sectional design, and thus is limited in its capacity to present causal relationships or to determine the direction of the relationship between alcohol consumption, cigarette smoking, metabolic syndrome and abnormal liver function. Second, we were not able to establish a diagnosis of AFLD or NAFLD. Instead we used AST, ALT, and GGT levels, which have been previously used as markers of AFLD or NAFLD, and restricted our analyses to participants who reported no liver disease or diabetes. In addition, some studies have suggested revised cut-off values for normal ALT levels (30 U/L for men and 19 U/L for women, or 35 U/L for men and 23 U/L for women instead of 40 U/L for both men and women which was applied in the present study) [46], [47], [48]. However, applying these different cut-off values in our dataset did not change the results, although there was a small difference in the magnitudes of risk (data not shown).

Third, there is the possibility of recall bias for at-risk behaviors such as cigarette smoking and alcohol consumption, particularly under-reporting in women, even though well-trained interviewers and a comprehensive, structured questionnaire were used. In addition, these at-risk behaviors can be influenced by socioeconomic characteristics, such as education level, average income, occupation, marital status, etc., or can influence metabolic syndrome [49], [50], [51]. In our analyses these influences were fully taken into account.

These limitations notwithstanding, the novel finding of this study is the observed combined risk of alcohol consumption, at levels lower than those previously accepted, and metabolic profile on liver damage. Alcohol consumption is an established risk factor for liver damage, and metabolic syndrome appears to be associated with elevated liver enzyme levels, which are accepted serological markers of AFLD and NAFLD [2], [19], [20]. However, so far little attention has been paid to the combined effect an alcohol consumption of <20–30 g/day and metabolic syndrome might have on liver damage, and this study shows a supra-additive effect of metabolic syndrome and heavy alcohol consumption (≥24 g/day), but also moderate alcohol consumption (<24 g/day), on liver damage. Furthermore, the present study suggested an effect of cigarette smoking either by itself, or combined with alcohol consumption on liver function, which is considerable as at present little evidences exists on the topic.

Given the rapid increase in the prevalence of adverse metabolic profiles, and the still high prevalence of alcohol consumption and cigarette smoking, our results embody an important public health message and suggest which strategies might be effective in preventing fatty liver disease and its progression to more severe liver diseases.
